# Poor sleep quality and overweight/obesity in healthcare professionals: a cross-sectional study

**DOI:** 10.3389/fpubh.2024.1390643

**Published:** 2024-05-30

**Authors:** Hongyun Huang, Tian Yu, Chengyu Liu, Jian Yang, Jianchun Yu

**Affiliations:** ^1^Department of General Surgery, Peking Union Medical College Hospital, Peking Union Medical College and Chinese Academy of Medical Sciences, Beijing, China; ^2^Department of General Surgery, Dongli District Traditional Chinese Medicine Hospital, Tianjin, China

**Keywords:** sleep quality, overweight and obesity, healthcare professionals, sleep duration, sleep disturbances

## Abstract

**Objective:**

This study aimed to analyze the relationship between the sleep quality of healthcare professionals and the incidence of overweight and obesity, exploring the potential impact of sleep quality on the onset of overweight and obesity in order to provide a scientific basis for formulating effective health intervention measures.

**Methods:**

A convenience sampling method was used to conduct a survey on the sleep characteristics and obesity status among healthcare professionals at Peking Union Medical College Hospital and Tianjin Dongli District Traditional Chinese Medicine Hospital. The survey was conducted via online questionnaires, which included demographic data, the Pittsburgh Sleep Quality Index (PSQI), height, weight, and related sleep, exercise, and dietary habits. Univariate and multivariate logistic regression analyses were applied to study the relationship between sleep quality and overweight/obesity among healthcare professionals.

**Results:**

A total of 402 questionnaires were distributed, with a 100% retrieval rate, yielding 402 valid questionnaires. The average body mass index of the 402 participants was 23.22 ± 3.87 kg/m^2. Among them, 144 cases were overweight or obese, accounting for 35.8% (144/402) of the total. The prevalence of poor sleep quality among healthcare professionals was 27.4% (110/402), with an average PSQI score of 8.37 ± 3.624. The rate of poor sleep quality was significantly higher in the overweight and obese group compared to the normal weight group (36.1% vs. 22.5%, *p* = 0.003). The multivariate analysis indicated that gender, marital status, lower education level, sleep duration (odds ratio [OR] =1.411, 95% confidence interval [CI] 1.043–1.910, *p* = 0.026), and sleep disturbances (OR = 1.574, 95%CI 1.123–2.206, *p* = 0.008) were significant risk factors for overweight and obesity among healthcare professionals.

**Conclusion:**

Overweight or obese healthcare professionals had poorer sleep quality compared to those with a normal weight. Sleep duration and sleep disorders were identified as independent risk factors for overweight or obesity in healthcare professionals. Increasing sleep duration and improving sleep disorders may play a positive role in controlling overweight and obesity among healthcare professionals.

## Introduction

Compared to the general population, healthcare professionals have been observed to exhibit a higher incidence of overweight and obesity (1, 2). A study investigating the prevalence of obesity among healthcare professionals in England demonstrated that healthcare staff, particularly nurses at a notable rate of 25.1%, experienced higher obesity rates compared to the general population (2). A survey of 4,241 Malaysian healthcare workers found that the overweight rate was 33.1% and the obesity rate was 21.1% (1). Sarı et al. (3) cross-sectional survey found 11.8% obesity and 37.8% overweight among healthcare workers. Shift work systems have been recognized as significant contributors to the heightened risk of overweight and obesity in healthcare professionals (4, 5). Studies have shown that shift workers are more than twice as likely to have a BMI of ≥25 compared to those in traditional work settings (6), with an increased duration of shift work correlating with a higher risk of being overweight or obese (7, 8). A large-scale epidemiological study demonstrated that every 5 years of rotating night shifts is associated with a 0.17 unit increase in BMI (95% CI 0.14–0.19) and a weight gain of 0.45 kg (95% CI: 0.38–0.53) (9).

For adults, a sleep duration of less than 7 h per night is classified as insufficient sleep (10). The rising prevalence of insufficient sleep, closely linked with various metabolic diseases, has emerged as a significant public health issue (11). Sleep problems are notably prevalent among healthcare professionals (12). Shift schedules in healthcare are known to disrupt normal circadian rhythms, resulting in chronic sleep deprivation and sleep disorders (13). Trockel MT et al.’s assessment of sleep-related disorders among 11,395 doctors revealed that 29.1% had sleep disorders, most prominently among surgical residents (14). Surveys indicate that more than one-third of medical students report sleeping less than 7 h (15). Insufficient sleep has been linked to professional burnout among healthcare professionals, with American Academy of Sleep Medicine (AASM) data revealing an approximately 50% prevalence of physician burnout (16).

Studies have suggested that insufficient sleep may be associated with overweight and obesity through changes in hormone levels and energy metabolism. Sleep restriction caused an increase in ghrelin levels and a decrease in leptin levels (17). In addition, sleep deprivation leads to increased energy intake and significant weight gain, especially in visceral fat (18). Based on these mechanistic findings, we hypothesized that poor sleep quality among healthcare professionals would increase the incidence of overweight or obesity. Therefore, we designed a questionnaire to investigate the sleep quality of healthcare professionals and explore its correlation with overweight and obesity.

## Materials and methods

### Study design, population, and sample

A cross-sectional survey was conducted among clinical healthcare professionals from Peking Union Medical College Hospital and Tianjin Dongli District Traditional Chinese Medicine Hospital. Clinical practitioners, including doctors and nurses, were included in the study, whereas non-clinical staff were excluded. The study used a convenience sampling method and was executed via an online questionnaire.

### Measures

The questionnaire included information such as height, weight, gender, age, marital status, educational level, occupation, and daily life habits of the participants. Weight and height were self-reported, and the body mass index (BMI) was calculated as weight (kg) divided by the square of the height (m^2^). According to the “Chinese Guidelines for Prevention and Control of Overweight and Obesity in Adults,” participants were categorized based on their BMI values into normal (BMI < 24 kg/m^2^) and overweight/obese (BMI ≥ 24 kg/m^2^) groups (19).

The Pittsburgh Sleep Quality Index (PSQI) (20, 21) was used to assess the sleep quality of healthcare professionals over the past month, including seven dimensions: sleep quality, sleep onset latency, sleep duration, sleep efficiency, sleep disturbances, use of sleeping medication, and daytime dysfunction. Each dimension had a scoring range from 0 to 3, with the sum of these dimensions constituting the total PSQI score. Higher scores indicated poorer sleep quality. In this study, a PSQI score of ≤10 was defined as good sleep quality, while >10 was defined as poor sleep quality.

### Statistical analysis

Data processing was carried out using SPSS 27.0 software. Quantitative data conforming to a normal distribution were represented as mean ± standard deviation. Non-normally distributed data were presented as medians (interquartile range). Count data were expressed in percentages (%). Intergroup comparisons were conducted using the chi-square test. Univariate logistic regression analysis was utilized to explore the relationship between various factors and overweight/obesity. Factors with a *p*-value of <0.05 were included in the multivariate logistic regression model analysis to analyze the association between sleep-related factors and overweight/obesity after adjusting for confounding factors, and variable selection was performed by the forward likelihood ratio method. A p-value of <0.05 was considered statistically significant.

## Result

### Prevalence of overweight and obesity among healthcare professionals

Out of the 402 questionnaires distributed, all 402 were successfully retrieved, resulting in a 100% response rate with valid responses. Among the healthcare professionals surveyed, 114 were from Peking Union Medical College Hospital and 288 from Tianjin Dongli District Traditional Chinese Medicine Hospital. Among 402 participants, 95 (23.6%) were men, 307 (76.4%) were women, and the median (interquartile range) age was 34 (14) years old. The average BMI was 23.22 ± 3.87 kg/m^2 and 35.8% (144/402) were overweight or obese. The prevalence of poor sleep quality was 27.4% (110/402), with an average PSQI score of 8.37 ± 3.624. The rate of poor sleep quality was significantly higher in the overweight and obese group compared to the normal-weight group (36.1% vs. 22.5%, *p* = 0.003). Statistically significant differences were observed between the overweight/obese group and the normal-weight group in terms of gender, region, marital status, age, and educational level (*p* < 0.05), whereas no significant differences were found in occupation, exercise, or dietary habits (Table 1).

**Table 1:** Characteristics of overweight and obesity among 402 healthcare professionals.

Characteristics	*N* (%)	BMI < 24	BMI ≥ 24	*p*-value
Gender				<0.001
Male	95 (23.6)	43 (16.7)	52 (36.1)	
Female	307 (76.4)	215 (83.3)	92 (63.9)	
Age	34 (14)	32 (14)	36 (14)	<0.001
Region				0.003
Beijing	114 (28.4)	86 (33.3)	28 (19.4)	
Tianjin	288 (71.6)	172 (66.7)	116 (80.6)	
PSQI				0.003
≤10	292 (72.6)	200 (77.5)	92 (63.9)	
>10	110 (27.4)	58 (22.5)	52 (36.1)	
Marital status				<0.001
Unmarried	136 (33.8)	107 (41.5)	29 (20.1)	
Married	256 (63.7)	147 (57.0)	109 (75.7)	
Other	10 (2.5)	4 (1.6)	6 (4.2)	
Educational level				<0.001
Undergraduate and below	270 (67.2)	157 (60.9)	113 (78.5)	
Graduate and above	132 (32.8)	101 (39.1)	31 (21.5)	
Occupation				0.720
Doctor	255 (63.4)	162 (62.8)	93 (64.6)	
Nurse	147 (36.6)	96 (37.2)	51 (35.4)	
Physical activity				0.069
Yes	128 (31.8)	74 (28.7)	54 (37.5)	
No	274 (68.2)	184 (71.3)	90 (62.5)	
Staying up late (times/week)				0.293
0	64 (15.9)	38 (14.7)	26 (18.1)	
1–2	191 (47.5)	130 (50.4)	61 (42.4)	
≥3	147 (36.6)	90 (34.9)	57 (39.6)	
Beverage consumption (times/week)				0.387
0	233 (58.0)	143 (55.4)	90 (62.5)	
1–2	66 (16.4)	45 (17.4)	21 (14.6)	
≥3	103 (25.6)	70 (27.1)	33 (22.9)	
Late night snacking (times/week)				0.288
0	243 (60.4)	154 (59.7)	89 (60.8)	
1–2	134 (33.3)	91 (35.3)	43 (29.9)	
≥3	25 (6.2)	13 (5.0)	12 (8.3)	

All values are expressed as *n* (%) except for age, which is expressed as the median (interquartile range).

PSQI, Pittsburgh Sleep Quality Index.

### Factors affecting sleep quality in healthcare professionals

The survey revealed that among overweight or obese healthcare professionals, the proportions of experiencing insomnia ≥3 times per week and voluntarily staying up late ≥3 times per week were 18.1% (26/144) and 39.6% (57/144), and 11.2% (29/258) and 34.9% (90/258) in the normal-weight group, respectively, but there was no significant difference between the two groups (*p* = 0.077 and *p* = 0.293). Among participants who were overweight or obese, 83.3% (120/144) attributed insomnia primarily to work or study. In terms of preventive measures for insomnia, the use of sleep medications was lower among those who were overweight or obese compared to those of normal weight (10.4% vs. 18.6%, *p* = 0.030). There was no significant difference between the two groups in the consumption of stimulating beverages, with 22.9% (33/144) of the overweight or obese individuals reporting needing these beverages ≥3 times per week, as compared to 27.1% (70/258) of the normal-weight individuals (*p* = 0.387) (Table 2).

**Table 2:** Factors influencing sleep quality in healthcare professionals.

Factors	N	BMI < 24 (%)	BMI ≥ 24 (%)	*χ*^2^ value	*p*-value
Frequency of Insomnia (times/week)				5.128	0.077
0	238	152 (58.9)	86 (59.7)		
1–2	109	77 (29.8)	32 (22.2)		
≥3	55	29 (11.2)	26 (18.1)		
Reasons for insomnia					
Work or study	347	227 (88.0)	120 (83.3)	1.693	0.193
Family issues	97	57 (22.1)	40 (27.8)	1.631	0.202
Emotional factors	59	40 (15.5)	19 (13.2)	0.394	0.530
Other	62	36 (14.0)	26 (18.1)	1.192	0.275
Frequency of staying up late (times/week)				2.454	0.293
0	64	38 (14.7)	26 (18.1)		
1–2	191	130 (50.4)	61 (42.4)		
≥3	147	90 (34.9)	57 (39.6)		
Reasons for staying up late^a^					
Work	227	142 (64.5)	85 (72.0)	1.953	0.162
Study	103	70 (31.8)	33 (28.0)	0.538	0.463
Entertainment	137	92 (41.8)	45 (38.1)	0.432	0.511
Family matters	68	41 (18.6)	27 (22.9)	0.861	0.353
Other	30	19 (8.6)	11 (9.3)	0.045	0.833
Demand for stimulant beverages (times/week)				1.899	0.387
0	233	143 (55.4)	90 (62.5)		
1–2	66	45 (17.4)	21 (14.6)		
≥3	103	70 (27.1)	33 (22.9)		
Measures to prevent insomnia					
Sleeping medication	63	48 (18.6)	15 (10.4)	4.688	0.030
Milk before sleep	29	18 (7.0)	11 (7.6)	0.061	0.806
Exercise	68	50 (19.4)	18 (12.5)	3.113	0.078
Foot bath	96	66 (25.6)	30 (20.8)	1.146	0.284
Traditional Chinese Medicine	21	13 (5.0)	8 (5.6)	0.050	0.823
Other	19	13 (5.0)	6 (4.2)	0.156	0.693

a Data analysis of reasons for staying up late was based on 338 subjects who stayed up late.

### Univariate analysis of overweight and obesity in healthcare professionals

The univariate logistic regression analysis revealed significant associations (*p* < 0.05) with eight factors: gender, age, region, PSQI total score, marital status, educational level, frequency of breakfast consumption, and snacking habits. Notably, a higher educational level (odds ratio [OR] =0.426, 95% confidence interval [CI] 0.267–0.682, *p* < 0.001) and lower frequency of snacking (less than three times/week) (OR = 0.587, 95% CI 0.376–0.916, *p* = 0.019) were inversely associated with overweight and obesity. Among the seven dimensions of the Sleep Quality Index, sleep duration (OR = 1.649, 95% CI 1.263–2.154, *p* < 0.001) and sleep disturbances (OR = 1.667, 95% CI 1.232–2.255, *p* < 0.001) emerged as significant risk factors for overweight and obesity among healthcare professionals (Table 3).

**Table 3:** Results of univariate logistic regression analysis for overweight and obesity among healthcare professionals.

Characteristics	*p* value	OR	95% CI
Gender (Female vs. Male)	<0.001	0.354	0.221–0.567
Age	<0.001	1.045	1.022–1.070
Region	0.003	2.071	1.273–3.372
PSQI (≥10 scores)	0.004	1.949	1.245–3.052
Marital status	<0.001		
Married vs. Unmarried	0.012	0.181	0.048–0.683
Other vs. Unmarried	0.284	0.494	0.136–1.794
Occupation	0.721	0.925	0.605–1.415
Physical activity	0.070	0.670	0.435–1.032
Educational level	<0.001	0.426	0.267–0.682
Breakfast (3times/week)	0.029	1.931	1.071–3.482
Snack intake (3times/week)	0.019	0.587	0.376–0.916
Fruit consumption (3times/week)	0.111	0.704	0.457–1.084
Water intake (>1,000 mL)	0.121	1.384	0.918–2.088
Late-Night snacking (3times/week)	0.194	1.713	0.760–3.862
Sleep quality	0.149	1.217	0.932–1.590
Sleep latency	0.514	1.074	0.867–1.330
Sleep duration	<0.001	1.649	1.263–2.154
Sleep efficiency	0.297	1.135	0.894–1.441
Sleep disturbances	<0.001	1.667	1.232–2.255
Use of sleeping medication	0.133	0.796	0.591–1.072
Daytime dysfunction	0.113	1.197	0.958–1.496

### Multivariate analysis of overweight and obesity in healthcare professionals

The results of the multivariate logistic regression analysis indicated that gender, marital status, educational level, sleep duration, and sleep disturbances were the principal factors influencing overweight and obesity among healthcare professionals. Upon controlling for factors such as gender, age, region, marital status, and educational level, shorter sleep duration (OR = 1.411, 95% CI 1.043–1.910, *p* = 0.026) and sleep disturbances (OR = 1.574, 95% CI 1.123–2.206, *p* = 0.008) were identified as independent risk factors for overweight and obesity among healthcare professionals (Table 4).

**Table 4:** Results of multivariate logistic regression analysis for overweight and obesity among healthcare professionals.

Characteristics	*p* value	OR	95% CI
Gender (Female vs. Male)	0.001	0.175	0.098–0.313
Age	0.685		
Region	0.874		
Marital status	0.007		
Married vs. Unmarried	0.067	0.260	0.062–1.098
Other vs. Unmarried	0.491	0.618	0.158–2.425
Educational level	0.002	0.395	0.222–0.704
Sleep duration	0.026	1.411	1.043–1.910
Sleep disturbances	0.008	1.574	1.123–2.206
Breakfast (≥ 3 times/week)	0.266		
Snack intake (≥ 3 times/week)	0.688		

## Discussion

Our study observed that 27.4% (110/402) of the surveyed healthcare professionals reported poor sleep quality, with a higher prevalence among those who were overweight or obese compared to their normal-weight counterparts (36.1% vs. 22.5%, *p* = 0.003). Additionally, reductions in sleep duration and the presence of sleep disturbances were significantly associated with an increased risk of overweight and obesity.

Patrick et al. conducted a large prospective study (*n* = 14,800) and found that each 0.50 standard deviation reduction in sleep duration was associated with a 1.45-fold increase in the probability of obesity and increased waist circumference (95% CI 1.03–2.04 and 1.02–2.06, respectively) (22). This finding highlights that insufficient sleep duration is an important factor in the increased risk of obesity and is consistent with our results. A survey by Nikfar et al. involving 552 hospital workers revealed that individuals with a lower BMI had better night-time sleep quality compared to those who were overweight or obese (23). In a meta-analysis by Itani et al., all of the 16 included studies were prospective cohort studies, which found that short sleep duration (<6 h/day) was associated with a significant increase in obesity and led to a 38% increase in absolute obesity incidence (OR = 1.38, 95% CI 1.25–1.53) (24).

Insufficient sleep is closely linked to overweight and obesity, potentially due to complex biological mechanisms, including hormonal regulation, genetic susceptibility, gut microbiome changes, and inflammatory responses. First, sleep can regulate body weight by affecting energy metabolism and levels of appetite-regulating hormones. Sleep deprivation has been shown to elevate the levels of the growth hormone-releasing peptide (ghrelin) and decrease the levels of leptin, thereby increasing hunger and energy intake (17). In a randomized controlled crossover study (*n* = 12) with sleep restricted to 4 h per night over 21 days, Covassin et al. discovered that reduced sleep duration significantly increased protein (*p* = 0.050) and fat (*p* = 0.046) intake, culminating in an additional daily caloric intake of 308 kcal (18). Notably, the study reported significant weight gain (*p* = 0.008) under conditions of sleep restriction, particularly in visceral fat accumulation (*p* = 0.042) (18). Second, genetic overlap exists between obesity and sleep-related genes, such as the circadian rhythm regulatory genes CLOCK and NR1D1, which are significantly associated with BMI and obesity-related traits (25–27). A Mendelian randomization study reveals that an increase of 1 h in sleep duration is associated with a 30% reduction in the risk of central obesity (OR = 0.70, 95% CI 0.64–0.77) (28).

Furthermore, the gut microbiome plays a key role in the relationship between sleep deprivation and obesity (29). Insufficient sleep increases gut permeability and bacterial translocation, leading to a reduced abundance of anti-obesity and anti-inflammatory microbial groups such as Bacteroidetes, Actinobacteria, and Bifidobacteria (30–32). Finally, sleep disorders are associated with increased markers of inflammation, potentially leading to the development of obesity (33, 34). A meta-analysis encompassing over 50,000 participants found that sleep disturbances are associated with elevated levels of CRP (effect size [ES] =0.12, 95%CI 0.05–0.19) and IL-6 (ES = 0.20, 95%CI 0.08–0.31) (35).

Additionally, gender, marital status, and education level significantly influence overweight and obesity among healthcare professionals. Female healthcare workers exhibit lower risk, likely due to enhanced social consciousness, proactive weight management strategies, and estrogen-induced increases in energy expenditure through the activation of brown adipose tissue and thermogenesis (36, 37). Conversely, married individuals may encounter heightened risk owing to increased meal frequency and portion sizes (38–40). Higher educational attainment is associated with enhanced health awareness, promoting improved dietary and exercise practices (41).

The study highlights the close relationship between the duration and quality of sleep and the risk of overweight and obesity. Consequently, adequate sleep is essential for maintaining a normal body weight, and effective sleep interventions are particularly crucial for improving the health status of healthcare professionals. Examples of such interventions include reducing the frequency and duration of night shifts (42), training in stress management and relaxation techniques (43), avoiding caffeine and nicotine intake, and optimizing the sleep environment (44).

### Strengths and limitations

This study presents novel findings by focusing on Chinese healthcare professionals, confirming the correlation between sleep disturbances and overweight/obesity, and emphasizing the impact of dietary habits, including breakfast consumption and snacking. The study has several limitations. Due to the retrospective data collection via questionnaire surveys, individual responses may not accurately reflect actual sleep patterns and weight management behaviors; the cross-sectional design precludes the determination of causal relationships; and the small sample size and geographical scope of the surveyed population restrict the comprehensiveness of the findings.

### Suggestions for future research

Future plans of the research team include utilizing objective sleep monitoring tools, such as sleep trackers or polysomnography, to provide more accurate data on sleep quality, duration, and patterns. Future studies could employ longitudinal study designs to observe the dynamic relationships over time among sleep patterns, work stress, and weight changes, thereby revealing causal relationships and providing a scientific basis for effective intervention strategies. Expanding the sample size and including healthcare professionals from various regions will facilitate the exploration of the impact of regional culture and work environments on sleep and obesity.

## Conclusion

Healthcare professionals who are overweight or obese tend to have poorer sleep quality compared to their normal-weight counterparts. The study reveals that reduced sleep duration and sleep disturbances significantly increase the risk of overweight and obesity among healthcare professionals. Moreover, extending sleep duration and ameliorating sleep disorders can play a beneficial role in managing overweight and obesity in this cohort.

## Data availability statement

The original contributions presented in the study are included in the article/supplementary material, further inquiries can be directed to the corresponding authors.

## Ethics statement

The studies involving humans were approved by the studies involving human participants were reviewed and approved by Ethics Committee of Peking Union Medical College Hospital. The studies were conducted in accordance with the local legislation and institutional requirements. Written informed consent for participation was not required from the participants or the participants' legal guardians/next of kin in accordance with the national legislation and institutional requirements.

## Author contributions

HYH: Data curation, Formal analysis, Investigation, Writing – original draft, Writing – review & editing. TY: Data curation, Resources, Software, Writing – review & editing. CYL: Methodology, Validation, Writing – review & editing. JY: Conceptualization, Project administration, Writing – review & editing. JCY: Conceptualization, Funding acquisition, Project administration, Supervision, Writing – review & editing.

## References

[1] Kunyahamu MS Daud A Jusoh N. Obesity among health-care workers: which occupations are at higher risk of being obese? Int J Environ Res Public Health. (2021) 18:4381. doi: 10.3390/ijerph18084381, PMID: 33924182 PMC8074354

[2] Kyle RG Wills J Mahoney C Hoyle L Kelly M Atherton IM. Obesity prevalence among healthcare professionals in England: a cross-sectional study using the health survey for England. BMJ Open. (2017) 7:e018498. doi: 10.1136/bmjopen-2017-018498, PMID: 29203505 PMC5719305

[3] Sarı H Kılınç Z Soysal Ş Özel M. Evaluation of the frequency and awareness of obesity among healthcare workers. Eur Rev Med Pharmacol Sci. (2023) 27:4639–47. doi: 10.26355/eurrev_202305_32475 37259748

[4] Tavares Amaro MG Conde de Almeida RA Marques Donalonso B Mazzo A Negrato CA. Prevalence of overweight and obesity among health professionals with shift work schedules: a scoping review. Chronobiol Int. (2023) 40:343–52. doi: 10.1080/07420528.2023.2174879, PMID: 36752069

[5] Saulle R Bernardi M Chiarini M Backhaus I La Torre G. Shift work, overweight and obesity in health professionals: a systematic review and Meta-analysis. Clin Ter. (2018) 169:e189–97. doi: 10.7417/T.2018.2077, PMID: 30151553

[6] Givens ML Malecki KC Peppard PE Palta M Said A Engelman CD. Shiftwork, sleep habits, and metabolic disparities: results from the survey of the health of Wisconsin. Sleep Health. (2015) 1:115–20. doi: 10.1016/j.sleh.2015.04.014, PMID: 26894229 PMC4755509

[7] Sun M Feng W Wang F Li P Li Z Li M. Meta-analysis on shift work and risks of specific obesity types. Obesity Rev. (2018) 19:28–40. doi: 10.1111/obr.12621, PMID: 28975706

[8] Smith KL Danyluk AB Munir SS Covassin N. Shift work and obesity risk-are there sex differences? Curr Diab Rep. (2022) 22:341–52. doi: 10.1007/s11892-022-01474-z, PMID: 35737274

[9] Pan A Schernhammer ES Sun Q Hu FB. Rotating night shift work and risk of type 2 diabetes: two prospective cohort studies in women. PLoS Med. (2011) 8:e1001141. doi: 10.1371/journal.pmed.1001141, PMID: 22162955 PMC3232220

[10] Chaput J-P Dutil C Featherstone R Ross R Giangregorio L Saunders TJ. Sleep duration and health in adults: an overview of systematic reviews. Appl Physiol Nutr Metab. (2020) 45:S218–31. doi: 10.1139/apnm-2020-0034, PMID: 33054337

[11] Liew SC Aung T. Sleep deprivation and its association with diseases- a review. Sleep Med. (2021) 77:192–204. doi: 10.1016/j.sleep.2020.07.048, PMID: 32951993

[12] Gr K Axelsson J. Health consequences of shift work and insufficient sleep. BMJ. (2016) 355:i5210. doi: 10.1136/bmj.i5210 27803010

[13] Stewart NH Arora VM. The impact of sleep and circadian disorders on physician burnout. Chest. (2019) 156:1022–30. doi: 10.1016/j.chest.2019.07.008, PMID: 31352036 PMC6859241

[14] Trockel MT Menon NK Rowe SG Stewart MT Smith R Lu M. Assessment of physician sleep and wellness, burnout, and clinically significant medical errors. JAMA Netw Open. (2020) 3:e2028111. doi: 10.1001/jamanetworkopen.2020.28111, PMID: 33284339 PMC12064096

[15] Johnson KM Simon N Wicks M Barr K O'Connor K Schaad D. Amount of sleep, daytime sleepiness, hazardous driving, and quality of life of second year medical students. Acad Psychiatry. (2017) 41:669–73. doi: 10.1007/s40596-017-0668-6, PMID: 28421480

[16] Kancherla BS Upender R Collen JF Rishi MA Sullivan SS Ahmed O. Sleep, fatigue and burnout among physicians: an American Academy of sleep medicine position statement. J Clin Sleep Med. (2020) 16:803–5. doi: 10.5664/jcsm.8408, PMID: 32108570 PMC7849815

[17] St-Onge MP. Sleep-obesity relation: underlying mechanisms and consequences for treatment. Obes Rev. (2017) 18:34–9. doi: 10.1111/obr.12499 28164452 PMC13098705

[18] Covassin N Singh P McCrady-Spitzer SK St Louis EK Calvin AD Levine JA. Effects of experimental sleep restriction on energy intake, energy expenditure, and visceral obesity. J Am Coll Cardiol. (2022) 79:1254–65. doi: 10.1016/j.jacc.2022.01.038, PMID: 35361348 PMC9187217

[19] Zhou B-F. Predictive values of body mass index and waist circumference for risk factors of certain related diseases in Chinese adults--study on optimal cut-off points of body mass index and waist circumference in Chinese adults. Biomed Environ Sci. (2002) 15:83–96. PMID: 12046553

[20] Buysse DJ Ancoli-Israel S Edinger JD Lichstein KL Morin CM. Recommendations for a standard research assessment of insomnia. Sleep. (2006) 29:1155–73. doi: 10.1093/sleep/29.9.1155 17040003

[21] Buysse DJ Reynolds CF Monk TH Berman SR Kupfer DJ. The Pittsburgh sleep quality index: a new instrument for psychiatric practice and research. Psychiatry Res. (1989) 28:193–213. doi: 10.1016/0165-1781(89)90047-4, PMID: 2748771

[22] Krueger PM Reither EN Peppard PE Burger AE Hale L. Cumulative exposure to short sleep and body mass outcomes: a prospective study. J Sleep Res. (2015) 24:629–38. doi: 10.1111/jsr.12327, PMID: 26211809 PMC5563465

[23] Nikfar B Moazzami B Chaichian S Ghalichi L Ekhlasi-Hundrieser M Chashmyazdan M. Sleep quality and its Main determinants among staff in a Persian private hospital. Arch Iran Med. (2018) 21:524–9. PMID: 30551693

[24] Itani O Jike M Watanabe N Kaneita Y. Short sleep duration and health outcomes: a systematic review, Meta-analysis, and Meta-regression. Sleep Med. (2017) 32:246–56. doi: 10.1016/j.sleep.2016.08.006, PMID: 27743803

[25] Locke AE Kahali B Berndt SI Justice AE Pers TH Day FR. Genetic studies of body mass index yield new insights for obesity biology. Nature. (2015) 518:197–206. doi: 10.1038/nature14177, PMID: 25673413 PMC4382211

[26] Lane JM Jones SE Dashti HS Wood AR Aragam KG van Hees VT. Biological and clinical insights from genetics of insomnia symptoms. Nat Genet. (2019) 51:387–93. doi: 10.1038/s41588-019-0361-7, PMID: 30804566 PMC6415688

[27] Dashti HS Daghlas I Lane JM Huang Y Udler MS Wang H. Genetic determinants of daytime napping and effects on Cardiometabolic health. Nat Commun. (2021) 12:900. doi: 10.1038/s41467-020-20585-3, PMID: 33568662 PMC7876146

[28] Liang YY Chen J Peng M Zhou J Chen X Tan X. Association between sleep duration and metabolic syndrome: linear and nonlinear Mendelian randomization analyses. J Transl Med. (2023) 21:90. doi: 10.1186/s12967-023-03920-2, PMID: 36747249 PMC9903442

[29] Withrow D Bowers SJ Depner CM González A Reynolds AC Wright KP Jr. Sleep and circadian disruption and the gut microbiome-possible links to dysregulated metabolism. Curr Opin Endocr Metab Res. (2021) 17:26–37. doi: 10.1016/j.coemr.2020.11.009, PMID: 34805616 PMC8597978

[30] Wang X Wang Z Cao J Dong Y Chen Y. Gut microbiota-derived metabolites mediate the neuroprotective effect of melatonin in cognitive impairment induced by sleep deprivation. Microbiome. (2023) 11:17. doi: 10.1186/s40168-022-01452-3, PMID: 36721179 PMC9887785

[31] Benedict C Vogel H Jonas W Woting A Blaut M Schürmann A. Gut microbiota and Glucometabolic alterations in response to recurrent partial sleep deprivation in Normal-weight young individuals. Mol Metab. (2016) 5:1175–86. doi: 10.1016/j.molmet.2016.10.003, PMID: 27900260 PMC5123208

[32] Pinart M Dötsch A Schlicht K Laudes M Bouwman J Forslund SK. Gut microbiome composition in obese and non-obese persons: a systematic review and meta-analysis. Nutrients. (2022) 14:12. doi: 10.3390/nu14010012, PMID: 35010887 PMC8746372

[33] Krysta K Krzystanek M Bratek A Krupka-Matuszczyk I. Sleep and inflammatory markers in different psychiatric disorders. J Neural Transm (Vienna). (2017) 124:179–86. doi: 10.1007/s00702-015-1492-3, PMID: 26649857 PMC5281642

[34] Huang Y Jiang Y Zhu M. The relationship between global sleep score and inflammatory markers in obese adults from the United States. Nat Sci Sleep. (2019) 11:317–24. doi: 10.2147/NSS.S220436, PMID: 31807104 PMC6842311

[35] Irwin MR Olmstead R Carroll JE. Sleep disturbance, sleep duration, and inflammation: a systematic review and Meta-analysis of cohort studies and experimental sleep deprivation. Biol Psychiatry. (2016) 80:40–52. doi: 10.1016/j.biopsych.2015.05.014, PMID: 26140821 PMC4666828

[36] Grzymisإéawska M EbA P Zawada A Grzymisإéawski M. Do nutritional behaviors depend on biological sex and cultural gender? *Adv*. Clin Exp Med. (2020) 29:165–72. doi: 10.17219/acem/111817, PMID: 32017478

[37] Mahboobifard F Pourgholami MH Jorjani M Dargahi L Amiri M Sadeghi S. Estrogen as a key regulator of energy homeostasis and metabolic health. Biomed Pharmacother. (2022) 156:113808. doi: 10.1016/j.biopha.2022.113808, PMID: 36252357

[38] Tzotzas T Vlahavas G Papadopoulou SK Kapantais E Kaklamanou D Hassapidou M. Marital status and educational level associated to obesity in Greek adults: data from the National Epidemiological Survey. BMC Public Health. (2010) 10:732. doi: 10.1186/1471-2458-10-732, PMID: 21110843 PMC3004837

[39] Sobal J Hanson KL Frongillo EA. Gender, ethnicity, marital status, and body weight in the United States. Obesity (Silver Spring). (2009) 17:2223–31. doi: 10.1038/oby.2009.64 19300431

[40] Song L Guan T Guo P Tan X Bryant AL Wood WA. Health behaviors, obesity, and marital status among Cancer survivors: a MEPS study. J Cancer Surviv. (2023) 17:499–508. doi: 10.1007/s11764-022-01269-x, PMID: 36409440 PMC10036458

[41] Li Y Cai T Wang H Guo G. Achieved educational attainment, inherited genetic endowment for education, and obesity. Biodemography Soc Biol. (2021) 66:132–44. doi: 10.1080/19485565.2020.1869919, PMID: 34182851 PMC8607810

[42] Zhang Y Murphy J Lammers-van der Holst HM Barger LK Lai Y-J Duffy JF. Interventions to improve the sleep of nurses: a systematic review. Res Nurs Health. (2023) 46:462–84. doi: 10.1002/nur.22337, PMID: 37710916 PMC10539041

[43] Ofei-Dodoo S Cleland-Leighton A Nilsen K Cloward JL Casey E. Impact of a mindfulness-based, workplace group yoga intervention on burnout, self-care, and compassion in health care professionals: a pilot study. J Occup Environ Med. (2020) 62:581–7. doi: 10.1097/JOM.0000000000001892, PMID: 32358474

[44] Baranwal N Yu PK Siegel NS. Sleep physiology, pathophysiology, and sleep hygiene. Prog Cardiovasc Dis. (2023) 77:59–69. doi: 10.1016/j.pcad.2023.02.005 36841492

